# ToPASeq: an R package for topology-based pathway analysis of microarray and RNA-Seq data

**DOI:** 10.1186/s12859-015-0763-1

**Published:** 2015-10-29

**Authors:** Ivana Ihnatova, Eva Budinska

**Affiliations:** Institute of Biostatistics and Analyses, Faculty of Medicine, Masarykova Univerzita, Brno, Czech Republic; Central European Institute of Technology, Brno, Czech Republic; RECETOX, Faculty of Science, Masarykova Univerzita, Brno, Czech Republic

**Keywords:** Topology, Pathway analysis, Microarray, RNA-Seq, Packages

## Abstract

**Background:**

Pathway analysis methods, in which differentially expressed genes are mapped to databases of reference pathways and relative enrichment is assessed, help investigators to propose biologically relevant hypotheses. The last generation of pathway analysis methods takes into account the topological structure of a pathway, which helps to increase both specificity and sensitivity of the findings. Simultaneously, the RNA-Seq technology is gaining popularity and becomes widely used for gene expression profiling. Unfortunately, majority of topological pathway analysis methods remains without implementation and if an implementation exists, it is limited in various factors.

**Results:**

We developed a new R/Bioconductor package ToPASeq offering uniform interface to seven distinct topology-based pathway analysis methods, of which three we implemented de-novo and four were adjusted from existing implementations. Apart this, ToPASeq offers a set of tailored visualization functions and functions for importing and manipulating pathways and their topologies, facilitating the application of the methods on different species. The package can be used to compare the differential expression of pathways between two conditions on both gene expression microarray and RNA-Seq data. The package is written in R and is available from Bioconductor 3.2 using AGPL-3 license.

**Conclusion:**

ToPASeq is a novel package that offers seven distinct methods for topology-based pathway analysis, which are easily applicable on microarray as well as RNA-Seq data, both in human and other species. At the same time, it provides specific tools for visualization of the results.

**Electronic supplementary material:**

The online version of this article (doi:10.1186/s12859-015-0763-1) contains supplementary material, which is available to authorized users.

## Background

High-throughput gene expression technologies (such as microarray or RNA-Seq) are used to estimate expression levels of thousands of genes in one experiment. Often the aim of such experiments is to find pathways and biological processes altered between two conditions, which helps investigators to propose biologically relevant hypotheses for further research. Achieving this aim implies integration of a priori known pathway information into the data analysis. Most often, a set of genes with similar biological function or participating in a regulatory process is employed as a set of entities in enrichment-based methods [1]. This approach, however, ignores known interactions between particular genes reflected in the topological structure. Thus, if a change in interactions occurs, this is not reflected in the results. The last generation of pathway analysis methods takes into account the topological structure of a pathway, which helps to increase both specificity and sensitivity of the findings.

Several types of methods for topology-based pathway analysis were proposed in the recent years (for review see [2]) - in all of them, the topological structure of a pathway is represented as graph with nodes (genes, proteins) and edges (interactions between genes/proteins). The methods test one of the two types of null hypotheses as proposed in [3] for gene set enrichment analysis. Independently on the hypothesis tested, we can further distinguish *multivariable* and *univariable* methods. For more detailed description of differences between multivariable vs univariable methods, we refer the reader to Additonal file 1.

Here, we focus on methods that (i) aim to identify pathways affected between two conditions based on differential expression of genes in the pathway - the most frequent aim of high-throughput genomic data studies, (ii) use the a priori known pathway topologies and (iii) use the pathway topologies separately.

The vast majority of existing topology pathway analysis methods were designed for continuous gene expression measures as obtained from microarray experiments. In order to apply them to discrete count data - a typical output from RNA-Seq experiment (number of reads mapped to a particular gene) - one must use a suitable transformation. Poisson or Negative binomial distribution are used as model distributions in differential expression analysis at gene-level for RNA-Seq data and a wide range of both transformation methods and statistical tests for this purpose exists. Performance of these methods is only recently being compared in extensive simulation studies [4–7].

The published methods are only rarely implemented as a publicly available software tool or package, and sometimes the existing implementation is not available anymore (e.g TAPPA [8]). The existing implementations can be divided into three categories: (i) commercial products (e.g. MetaCore [9]); (ii) R-packages (e.g. SPIA [10]) (iii) standalone applications (e.g. PWEA [11] or PRS [12]) and (iv) web-based applications (e.g. iPathwayGuide [13]). All of these tools usually offer embedded pathway topologies with a limited battery of methods (typically only one) and simple visualization (if any) of the results. Simultaneous application of different methods and comparison of their results is therefore very time-consuming, cumbersome and prone to clerical errors due to need for repeated data conversion and transfer. Additionally, the results may not be directly comparable, since some of the implementations use either built-in pathway topologies or their own pathway topology processing algorithm that leads to different topological structures. One of the best existing tools offering common interface to four topology-based pathway analysis methods (TopologyGSA [14], clipper [15], DEGraph [16] and SPIA [17]) is the R/Bioconductor package graphite [18]. The user can also access lists of parsed pathway topologies for some of the most common experimental organisms (14 in version 1.14.1) from several distinct databases (up to 6 for H. Sapiens, same version) stored as objects of class PathwayList where individual pathways are represented as instances of class Pathway. Although more pathways can be obtained from public databases or specialized websites and parsed to the R environment with available CRAN/Bioconductor packages, there is no transformation function from other pathway classes to the PathwayList or Pathway. The current graphite implementation has no uniform way of calling methods or specification of their parameters, making simultaneous application of different methods unhandy. Additionally, SPIA is limited only to data with EntrezGene identifiers and the signs of the interactions are neglected in DEGraph.

Here, we present ToPASeq (Topology-based Pathway Analysis of microarray and RNA-Seq data) - a Bioconductor package that adjusts the set of methods available through graphite and extends them by addition of three more methods. The package offers their unified manipulation and provides tools for their easy application on RNA-Seq count data. In addition, it provides special functions for conversion of user-imported pathways into Pathway class and a set of tools for coercing graphs between different formats and manipulation and visualization of the results.

In section Implementation, we describe the software implementation and available functions. Concrete examples of package usage and its comparison to other tools are given section Results and Discussion.

## Implementation

ToPASeq was implemented using statistical programming language R and the package is available through the open-source Bioconductor project [19].

In order to apply a topology-based pathway analysis method we need (i) gene expression measurements (a gene expression data matrix in which rows refer to genes and columns to samples), (ii) a vector with sample class labels and (iii) a list of pathways of interest together with their topologies in a specific format. The gene expression measurements and sample class information are usually available from the experiment.

### Pathway topologies and their manipulation

Pathway topologies are necessary for topology-based pathway analysis and can be created manually, or - even better - obtained from public databases or R packages, where they are typically stored in one of the standardized formats (KGML, BioPax, specific R classes). These formats, however, need to be parsed (downloaded and converted to specific format) to be used within the methods’ particular implementations. Within R framework, multiple ways exist for pathway topology/graph representation. More detailed description of some of them in the context of biological pathways can be found in Additional file 1.

Our package requires the pathway topologies in format defined as S4 class PathwayList where individual pathways are of class Pathway, which allows combination of oriented and not-oriented edges as well as multiple edges between nodes. We have especially designed several transformation functions that convert the most common formats into Pathway.

The users might be interested in manual editing of topology of the parsed pathways. We added group of methods such as (i) adding/removing of the nodes and edges, (ii) changing the type of interaction/directionality, (iii) merging two pathways into one, (iv) obtaining the induced subgraph. Additionally, the user may need to select only a subset of pathways based on their topological properties (e.g. number of edges related to a particular node, number of nodes, number of edges, number of connected components etc.). These can be easily obtained with other set of available functions.

Moreover, we especially designed a new function reduceGraph which merges the user defined named sets of nodes into a single node. The members of the sets must form either a gene family or a protein complex. The another function estimateCF estimates the maximal list of the sets of the nodes that can be merged. Finally, we provide a general function convertIdentifiersByVector which requires user specified information. For the detailed desctiption of the functionalities mentioned above we refer the reader to Additional file 1.

### Methods for topology-based pathway analysis

The package offers seven different methods covering various approaches in topological pathway analysis (see Table 1 for details). For detailed description of each method the reader is referred to cited references. We will focus on those aspects that are relevant to methods’ new implementation. All methods are implemented as a single function that applies the method over the list of pathways. More detailed description of differences between previous implementations of methods to our implementation can be found in Additional file 1.

**Table 1:** Methods included in the package

Method	Ref.	Type ^*a*^	Hypothesis	A/I ^*b*^	Primary Graph	Implementation	Input data ^*c*^
TopologyGSA	[14]	M	self-contained	No	DAG	adjusted	GEDM
DEGraph	[16]	M	self-contained	Yes	DAG	adjusted	GEDM
clipper	[15]	M	self-contained	No	DAG	adjusted	GEDM
SPIA	[17],	U	competitive	Yes	directed	adjusted	DEG and their log fold-change
	[25]						
PRS	[26]	U	competitive	No	directed	de novo	DEG and their log-fold change
PWEA	[27]	U	competitve	No	undirected	de novo	gene-level statistics
TAPPA	[8]	U	self-contained	No	undirected	de novo	GEDM

^*a*^ - M - multivariable, U - univariable ^*b*^ - A - Activation, I - Inhibition ^*c*^ - the data related to the pathway topology

We imported and adjusted the implemetation of the following methods: TopologyGSA, DEGraph, SPIA and Clipper. We found that the original implementation of the TopologyGSA method is extremely computationally intense for some of the pathways as the authors employ function that implements the exact branch-and-bound algorithm [20] to detect all of the cliques (subsets of nodes where every two nodes are connected by an edge) in a pathway topology. In our implementation, we substituted this function with getCliques which implements more efficient Bron-Kerbosch algorithm [21]. For the DEGraph method we have created a new wrapper function that preserves the possibility to consider interaction types (activation and inhibiton) and transforms the results into more user-friendly format - a data frame. The previous implementations of the SPIA method were limited to Entrez identificators. In our package we have bypassed this limitation by incorporating a more general converting function. Additionally, the user can also obtain a gene-level net perturbation accumulation — a measure of the importance of a gene in the topology. The Clipper method constists of two steps: (i) first, the differential expression of a pathway is assessed, (ii) then, the pathway topology is transformed into a junction tree and the portions of the tree which are mostly associated with phenotype are identified. We designed a new function that performs both steps of the algorithm in a single call.

In all of the imported and adjusted implementations we also added, when appropriate, an additional parameter specifying how should be the undirected interactions oriented. The user can choose whether an edge is oriented in both directions or only in one according to the order of the nodes.

We de-novo implemented three methods: TAPPA, PWEA, PRS, for which there was no implementation available within R framework. The PRS and PWEA are implemented in MATLAB and C++ respectively and these tools are discussed in the section Comparison with other tools. Our de-novo implementations are settled for pathway topologies from graphite package where one node is represented by only one gene or protein. Both PWEA and PRS methods incorporate a permutation-based test in order to assess the statistical significance of the pathway score. Considering the computational complexity of this approach we parallelized the crucial step of the PWEA method (repeated application of the differential expression analysis). In addition, the function for obtaining the number of the differentially expressed genes in PRS algorithm was implemented in C++ via Rcpp package.

**Fig. 1:**
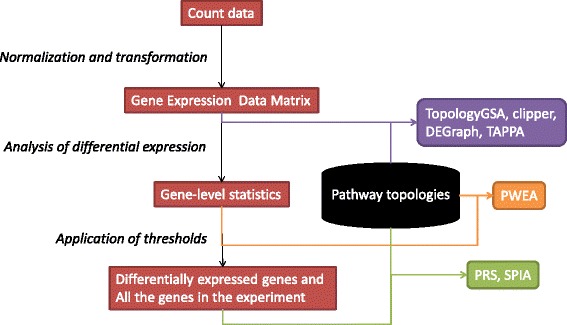
Schema of a processing pipeline. The red boxes refer to the outputs from regular analysis of differentially expressed genes and possible inputs for topology-based pathway analysis. Arrows indicate the processing pipeline of each of the methods implemented in the package

While several methods (TopologyGSA, DEGraph, Clipper and TAPPA) work directly with normalized gene expression values, others (SPIA, PRS and PWEA) use the result of differential gene-expression analysis with or without application of significance thresholds to obtain the list of differentially expressed genes (Fig. 1). With respect to this, all the methods were adapted also for a simple use of RNA-Seq count data. First, we employed pre-processing step for RNA-Seq normalization, with a selection of two best performing methods TMM [22], DESeq [23], as compared in Dillies et al. [4] and regularized log transformation from DESeq2 package which effectively removes the mean-variance relationship known in RNA-Seq data. Second, we added methods for RNA-Seq differential gene expression analysis (from limma and DESeq2 packages).

### Usage and visualization

Each method is implemented as a single wrapper function which allows the user to call a method in a single command. The wrapper function offers: (i) normalization of count data; (ii) differential gene expression analysis and (iii) pathway analysis. The data input types were unified for all the methods. Expression data can be supplied both as matrix or as ExpressionSet. The functions’ outputs have uniform format defined as a new S3 class topResult with specified output of generic functions (print, plot, summary) and methods for accessing individual slots of the resulting object. The users can specify which method should be used for normalization or differential expression analysis of the RNA-Seq data, with respect to their own preferences. This data pre-processing step can be completely omitted and users can submit already normalized data or, if appropriate, the results of the differential expression analysis (a table containing log fold-changes, statistics and *p*-values). Note, that PWEA method requires also so called Topology Influence Factors (TIFs), which need to be calculated from normalized gene expression data matrix.

**Fig. 2:**
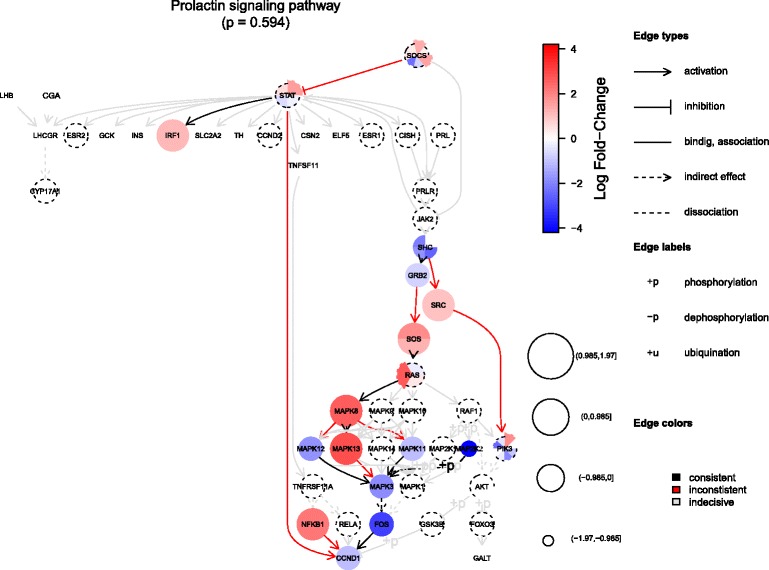
Visualization of the results after merging some of the gene families into one node. Some of the genes families present in the pathway were merged into single nodes. Those nodes are drawn as pie-chart, in which the number of slices equals to the number of gene merged. The colour, border and radius are preserved from the complete graph (Fig. 2 in Additional file 1). Average log fold-change is used as representative value, when the agreement between expression and interaction type is assessed

When the generic function plot() is applied to a topResult class, together with a name of the pathway or position in the list of pathways identifying the pathway to be plotted, a visualization of the pathway with three gene-level statistics is produced (Fig. 1 in Additional file 1. The user can specify a threshold by which an agreement between the expression status of the nodes and the interaction type between them is examined (Fig. 2 in Additional file 1).

The topology can be reduced by user specified list of nodes that are to be merged into one node. In this situation a pie chart is used as a representation of a node and the number of slices equals to the number of nodes merged. The filling colour and the radius is preserved from the separated nodes (Fig. 2). By default a mean change of the gene expression is used as a representative of the values when the agreement between gene expression and the interaction type is examined, but the user can specify another aggregation function. A slightly modified graph is plotted for TopologyGSA and Clipper, which perform differential expression analysis of the cliques. Since a single node can be a member of more than one clique, the colour of edges is used for their visualization (Fig. 4 in Additional file 1).

## Results and Discussion

For a simple example of how to create and manipulate a pathway, we refer the reader to Additional file 1.

We provide a simple application example of implemented methods on a RNA-Seq dataset. For more detailed descriptions of all the functions we refer the reader to the package manual.

The aim is to compare gene expression profiles between wild-type and RNA-binding protein hnRNP C (HNRNPC) knockdown HeLa cells [24]. The RNA-Seq dataset came from gageData package. There are four knockdown samples and four experimental samples in this dataset containing the count data for 22932 genes. We load the data and remove genes with count 0 in all samples:



... download the KEGG pathways and apply all seven topology-based pathway methods:



The arguments of all functions are as follows (from left to the right): a count matrix (or gene expression data matrix), a grouping vector, list of pathways with topologies and a type of the data). The TMM normalization and the limma-based differential gene-expression analysis are used by default. The pre-set thresholds for considering a gene significant are *p*-value less than 0.05 and the absolute log fold change above 2. Further, the gene identifiers in pathways are automatically converted to the EntrezGene identifiers and the non-oriented edges are oriented in both directions, when required.

The results for an individual pathway can be visualized as shown in Fig. 1 in Additional file 1:



### Comparison with other tools

The known previous implementations of the methods (if any) offered in ToPASeq are summarized in Table 2. We will further discuss only the methods implemented de-novo in R/Bioconductor frame work. For TAPPA there is no other available implementation known to the authors. A C++ implementation of PWEA can be downloaded from http://zlab.bu.edu/PWEA/download.php. The expression data have to be in the GSD format from Gene Expression Omnibus, where the probesets are named by both manufacturer IDs and the gene symbols. It is coupled with python script for retrieving and processing of KEGG.xml and.gene files. Beside the limitation to KEGG pathways and the need for manual downloading of non-human pathways or conversion to KGML format, it can be run only on UNIX-like systems. Recently, a standalone MATLAB-based implementation of PRS was published [12]. The application requires normalized microarray data in XLS file with manufacturer identifiers of the probesets, together with specification of the platform and the normalization method that was applied to the data. The set of possible platforms is limited to selection of Affymetrix HG and one Agilent platform. The user has no control over the pathway topologies that are used.

**Table 2:** Known implementation of the methods provided in ToPASeq

Method	Language	Source	Pathways	Format	Input data	Methods	Issusses
topologyGSA	R	Bioconductor	one example	graphNEL	GEDM	topologyGSA	too computationaly intense
clipper	R	Bioconductor	imported from graphite	pathway	GEDM	clipper	two separate steps necessary
DEGraph	R	Bioconductor	parsing function for KGML	graphNEL	GEDM	DEGraph	
SPIA	R	Bioconductor	parsing function for KGML, H. sapiens and M. musculus pre-parsed	list of adjacency matrices	DEG and log fold-changes	SPIA	Only for EntrezGene IDs
PRS tool	MATLAB	web ^*a*^	KEGG	unknown	GEDM	PRS	can not add or modify pathways, the data must have manufacturer probeset IDs, limited set of: possible platforms, DE tests,
PWEA	C++	web ^*b*^	human pathways from KEGG	unknown	GSD	PWEA	only for UNIX-like
TAPPA	Java	web ^*c*^	KEGG or PPI added to a gene set	-	-	TAPPA	not available
graphite	R	Bioconductor	pathways for 14 species from up to 6 databases	Pathway	depends on the method	topologyGSA, clipper, SPIA, DEGraph,	suboptimal import of the methods

^*a*^ - http: //www.buckingham.ac.uk/research/clore-laboratory-diabetes-obesity-and-metabolic-research/staff/maysson-al-haj-ibrahim/prs-tool/ ^*b*^ - http://zlab.bu.edu/PWEA/index.php ^*c*^ - http://watson.mcgee.mcw.edu:8080/~sgao, the page is down. (First accessed 4 Apr 2012) PPI - protein-protein interactions GEDM - gene expression data matrix, log2-transformed and normalized expression profiles

None of these tools allows for different method for normalization (e.g normalization with custom CDF-files from http://brainarray.mbni.med.umich.edu) or differential expression analysis; nor can it be used to analyse the RNA-Seq data.

Some users may prefer Cytoscape for visualization of pathways, since it provides user-friendly and interactive interface, which can be achieved using the RCytoscape package. Within this interface, however, the user can specify only the basic graphical parameters like size, shape or colour of the nodes or the styles of edges. Advanced graphical approaches provided through plug-ins can be accessed only directly from Cytoscape. We are currently working on the option of interactive graph visualization.

## Conclusions

Topology-based pathway analysis comprises a new generation of methods in gene set analysis, with the potential of generating more sensitive and more specific results. Currently, high-throughput technologies producing gene expression data that serve as input to these methods are employed in almost every biological and biomedical research with RNA-Seq being in the leader position. Tools for comfortable and quick application of these methods and visualization of their results are needed. Available packages or standalone applications are usually limited to one or few methods, readily applicable mainly to human studies and rarely contain also a visualization tool. We propose ToPASeq, a Bioconductor package providing a set of easy-to-use and general tools for topology-based pathway analysis within the R workspace. It offers seven distinct topology-based pathway analysis methods that cover wide range of approaches and can be easily applied on both microarray and RNA-Seq data. It also offers a visualization tool that is able to capture all the relevant information about the expression of genes within one pathway. Finally, the functions for pathway conversion extend the application of topology-based pathway analysis to experiments on species other than human.

## Availability and requirements

Project name: ToPASeq Project home page: http://www.bioconductor.org/packages/release/bioc/html/ToPASeq.html Operating system(s): Platform independent Programming language: R Other requirements: R version 3.2.1, CRAN and Bioconductor packages: graphite (>= 1.14), graph, gRbase License: AGPL-3 Any restrictions to use by non-academics: none Availability of supporting data: EBI ArrayExpress Experiment E-MTAB-1147: http://www.ebi.ac.uk/arrayexpress/experiments/E-MTAB-1147/, also in gageData package

## Additional file

Additional file 1 **Supplementary material.pdf.** The file contains additional details on the following: i) common principles of the multivariable and univariable topology-based methods; ii) the functions for pathway creation and manipulation (desciption as well as demostration); iii) comparison of ToPASeq with existing tools. (1013 Kb)
