# Supplementary material for: ToPASeq: an R package for topology-based pathway analysis of microarray and RNA-Seq data
Source: BMC Bioinformatics. 2015 Oct 29;16:350. doi: 10.1186/s12859-015-0763-1 (PMC4625615; doi:10.1186/s12859-015-0763-1)
Supplement: Supplementary file 1 — Supplementary material.pdf. The file contains additional details on the following: i) common principles of the multivariable and univariable topology-based methods; ii) the functions for pathway creation and manipulation (desciption as well as demostration); iii) comparison of ToPASeq with existing tools. (1013 Kb) [file 12859_2015_763_MOESM1_ESM.pdf]

Details on multivariable and univariable methods

The topology-based pathway analysis methods test one of the two types of null hypotheses as proposed in [6] for gene set enrichment analysis. The first hypothesis expects the genes in a pathway to be at most as often differentially expressed as the genes outside the pathway (the remaining genes measured in the experiment). Methods testing this hypothesis typically use gene randomization in the assessment of statistical significance and cannot be applied in an experiment that only measured expression of genes in a particular pathway. The second group of methods tests the hypothesis that no gene from a pathway is differentially expressed. These methods can be used for both genome-wide as well as pathway-specific experiments but require sufficient sample size for sample randomization. Independently on the hypothesis tested, we can further distinguish *multivariable* and *univariable* methods. Known multivariable methods either use Gaussian Graphical Models [9] or Fourier analysis on graphs [8]. Univariable methods, typically increase the weight of the differentially expressed genes as function of their topological properties (position in the graph, proximity of other differentially expressed genes etc.) [7, 1, 12] or transform the expression profile of each sample separately from gene-level to pathway-level [5]. As a consequence, a change in the topological structure influences the result of the analysis in any of the topology-based methods.

Pathway creation and manipulation

In topology-based pathway analysis, pathways are represented as graphs $G = (V, E)$, where V denotes a set of vertices or nodes represented by genes (or rather their functional products - proteins) and $E \subseteq V \times V$ is a set of edges between nodes (oriented or not, depending on the method) representing interactions between genes.

Within R framework, multiple ways exist for pathway topology / graph representation. The simplest is to use adjacency matrix - a mathematic representation of a graph as a $n \times n$ matrix $A = \{a_{ij}\}$, where n equals to the number of nodes and $a_{ij} = 1$ if there is an edge from node i to node j and $a_{ij} = 0$ otherwise. The matrix is symmetrical for undirected graphs. The most used format, however, is `graphNEL` class for which many manipulation, conversion and visualization tools are available. This class is at the same time used by some of the original implementations of topology-based pathway analysis methods.

Biological pathways, however, are more complex, and contain two special types of multi-gene nodes: a multi-subunit protein complex and a gene family. An interaction between all subunits of a protein complex is assumed, but members of a gene family are believed to be interchangeable. In addition, non-protein chemical compounds which cannot be measured in the gene expression experiments are also present in biological pathways. The parsing algorithms tend to eliminate these unmeasured compounds, creating thus corrupted pathway topologies. The authors of the `graphite` package therefore propose an approach in which the signal is propagated through such a compound mediated interactions and define a new class for pathway topology representation `Pathway`. This class, in addition, allows combination of oriented and not-oriented edges and more than one edge between two nodes. However, the pathway may not contain any unconnected nodes. `graphite` also provides the largest collection of parsed pathways.

If the user needs to prepare/parse pathways into `Pathway` class manually, our package provides multiple specific conversion functions: (i) `graphNEL2Pathway` function can be used for conversion from `graphNEL`; (ii) `AdjacencyMatrix2Pathway` function for conversion from an adjacency matrix; (iii) `KEGG2Pathway` function can be used to directly parse a KEGG pathway to `Pathway`. In order to create `graphNEL` one can use the `rBiopaxParser` package to parse from BioPax format. In addition, `rBiopaxParser` provides an option for conversion of pathways stored in BioPax format into extended adjacency matrix, which discriminates the type of the interaction: 1 refers to activation or neutral interaction and -1 is used for the inhibitory interaction. These conversion functions, however, neither expand the multi-subunit protein complexes nor propagate the signal through compound mediated interactions, since in `graphNEL` and adjacency matrix, this information is lost. On the other hand, the `KEGG2Pathway` has an advantage of complete pathway description available. Therefore with this function, the user can specify wheter the multi-subunit protein complexes should be expanded into cliques and/or the signal should be propagated through non-protein coding nodes. We are currently working on similar direct conversion tool for BioPax.

The users might be interested in manual editing of topology of the parsed pathways. We added group of methods defined in the `graph` class (a virtual class that all graph classes should extend) and functions from

graph package such as (i) adding/removing of the nodes (addNodes, removeNodes) and edges (addEdges, removeEdges), (ii) changing the type of interaction/directionality (changeInteraction, changeDirection), (iii) merging two pathways into one (join, union), (iv)obtaining the induced subGraph (subGraph). Additionally, the user may need to select only a subset of pathways based on their topological properties (e.g. number of edges related to a particular node, number of nodes, number of edges, number of connected components, nodes without any edge etc.). These can be easily obtained with other available functions such as: degree, numNodes, numEdges, connComp, numNoEdges etc.

The expansion of the gene family members and the protein complexes is useful for the topology-based pathway analysis as it provides complete information about all interactions a particular gene is involved in or its position in the pathway. However, as the number of edges increases with this expansion, the readability of the graph decreases due to too many crossing edges. Therefore, we especially designed a new function reduceGraph which merges the user defined named sets of nodes into a single node. It requires that the members of a set have either the same outgoing and/or incoming edges (they represent a gene family) or have undirected binding interaction between each other (they are the subunits of one protein complex). The user can use the function estimateCF to obtain the maximal list of the sets of genes that can be merged. The function attempts to recover the representative name as the common part of the individual gene names (e.g. a gene set containing AKT1, AKT2, AKT3 is named AKT). Since this may lead to ambiguities or missings, a manual correction of the recovered representative names may be needed. Finally, the function convertIdentifiers from the graphite package uses the data provided by the Bioconductor package org.Hs.eg.db for mapping node labels to either EntrezIDs or gene symbols. Since it is restricted to only human genes and may also lead, without warning, to the loss of the genes with unrecognized identifiers and consequently to corruption of the topology, we provide a general function convertIdentifiersByVector which requires user specified information.

With a simple example we will demonstrate how to create a pathway from an adjacency matrix. First we create the adjacency matrix.

```
> genes<-paste("gene", 1:5, sep="")
> adjmat<-matrix(sample(c(0,0,0,0,1), 25, TRUE),
+ 5,5, dimnames=list(genes,genes))
> adjmat
      gene1 gene2 gene3 gene4 gene5
gene1     0     1     0     0     1
gene2     0     0     1     0     1
gene3     0     1     1     0     0
gene4     0     0     0     1     1
gene5     0     0     0     0     0
```

Then, we apply AdjacencyMatrix2Pathway function from ToPASeq.

```
> p<-AdjacencyMatrix2Pathway(adjmat)
> p
"pathway" pathway
Native ID           = pathway
Database            = unknown
Species             = unknown
Type of identifiers = unknown
Number of nodes     = 5
Number of edges     = 8
Retrieved on        = 07-09-2015
```

We will use another example (the Complement cascade pathway from Reactome) to demonstrate two functions for identifier conversion. We can either use the automatic graphite function convertIdentifiers or convert the identifiers manually using a custom vector of identifiers and function convertIdentifiersByVector. In our example, we will convert Uniprot IDs to gene symbols. We extract the pathway:

```
> g<-pathways("hsapiens", "reactome")[["Complement cascade"]]
>  g
"Complement cascade" pathway
Native ID           = REACT_6932
Database            = Reactome
Species             = hsapiens
Type of identifiers = UNIPROT
Number of nodes     = 194
Number of edges     = 7378
Retrieved on        = 03-04-2015
```

...and perform the automatic conversion:

```
>ga<-convertIdentifiers(g,"symbol")
> ga
"Complement cascade" pathway
Native ID           = REACT_6932
Database            = Reactome
Species             = hsapiens
Type of identifiers = SYMBOL
Number of nodes     = 38
Number of edges     = 225
Retrieved on        = 03-04-2015
```

Please note, that the pathway now contains only 38 nodes. All the nodes that could not be converted are lost.

For the manual conversion, we first create a character vector of new identifiers with attribute names corresponding to the original UniProt identifiers. We will convert only three nodes out of 194. This demonstrates the advantage of manual conversion, which can be applied only on selected subset of nodes, which is advantageous, if the new identifiers are not available for all the nodes.

```
> genenames<-c("IGLC7", "IGHV", "IGLV")
> conv<-setNames(genenames, c("UniProt:A0M8Q6", "UniProt:A2KUC3", "UniProt:A2NXD2"))
```

Once the named vector is ready, we apply the converting function and check the nodes of the pathway:

```
> genenames<-c("IGLC7", "IGHV", "IGLV")
>  conv<-setNames(genenames, c("A0M8Q6", "A2KUC3", "A2NXD2"))
>
> gc<-convertIdentifiersByVector(g, conv, "gene symbol")
Warning message:
In convertIdentifiersByVector(g, conv, "gene symbol") :
  These pathway nodes are missing in the 'conv.table': P01609,
   P04207, P01825, Q6PIL0, P01719, P01771, P02746, P01777 [... truncated]

> head(sort(nodes(gc)))
[1] "A2NJV5" "IGHV"   "IGLC7"  "IGLV"   "O00187" "O00602"
> gc
"Complement cascade" pathway
Native ID           = REACT_6932
Database            = Reactome
Species             = hsapiens
Type of identifiers = gene symbol
Number of nodes     = 194
Number of edges     = 7378
Retrieved on        = 03-04-2015
```

On another example (the Prolactin signaling pathway from KEGG database) we will demonstrate how to merge genes from the same gene family into one node (compare Figure 1 and Figure 3). The user has to specify a list of genes to be reduced. This can be done either manually:

```
> g<-pathways("hsapiens","kegg")[["Prolactin signaling pathway"]]
> reduction_manual<-list(RAS=c("NRAS","KRAS","HRAS"),
+ SHC=c("SHC1", "SHC4","SHC2","SHC3"),
+ AKT=c("AKT1","AKT2","AKT3"),
+ STAT=c("STAT5B","STAT3","STAT5A"),
+ SOCS=c("SOCS2","SOCS5", "SOCS7","SOCS1",
+ "SOCS6","SOCS4", "SOCS3"),
+ PIK3=c("PIK3R3", "PIK3R1", "PIK3CA",
+ "PIK3R5","PIK3CD", "PIK3R2","PIK3CB",
+ "PIK3CG"), MAPK=c("MAPK12","MAPK11",
+ "MAPK13", "MAPK14"))
> reduced<-reduceGraph(g, reduction_manual)
```

...or the user can use the `estimateCF` function. Since there are no protein complexes present in this pathway, we will merge only a subset of gene families:

```
> reduction_auto<-estimateCF(g)[[2]]
> reduction_auto<-reduction_auto[c(1,3,5,6,7,12,13)]
# or for the maximal subset with recovered representative names
# reduction_auto<-reduction_auto[!duplicated(names(reduction_auto)) &
# regexpr("family", names(reduction_auto))==-1)]
> reduced<-reduceGraph(g, reduction_auto)
```

Methods for topology-based pathway analysis

The package offers seven different methods covering various approaches in topological pathway analysis, of which four were imported and adjusted from other packages and three were de-novo implemented in R (see Table 1 for details). For detailed description of each method the reader is referred to cited references. We will focus on those aspects that are relevant to methods' new implementation.

The TopologyGSA was first implemented in the `topologyGSA` package and is available through the `graphite` as either `runTopologyGSA` or `runTopologyGSAMulti` for one or a list of pathways, respectively. Both implementations are extremely computationally intense for some of the pathways as they employ function that implements the exact branch-and-bound algorithm [10] to detect all of the cliques (subsets of nodes where every two nodes are connected by an edge) in a pathway topology. In our implementation, we substituted this function with `getCliques` which implements more efficient Bron-Kerbosch algorithm [3].

Another Bioconductor package `DEGraph` provides implementation of DEGraph method [8]. The user can apply this method either on one or all connected components of a single pathway with `testOneComponent` and `testOneGraph`, respectively. The user can decide, whether the method should consider the type of the interaction (e.g. activation or inhibition). The `testOneGraph` function is called within a wrapper function from `graphite` with analogous name `runDEGraph` and `runDEGraphMulti`. However, the type of the interaction is explicitly suppressed - this is in contrast to the complexity of the pathway topologies in this package. In our implementation we preserve the possibility to decide on the consideration of the interaction types.

The `SPIA` Bioconductor package offers implementation of the SPIA method. This method represents an approach with the most detailed view on the interaction types. The method is applied on KEGG pathways as follows. First, one needs to download the pathway topologies in KGML format and parse them into a SPIA-specific format. The pathways of *H. sapiens* and *M. musculus* are directly available. Second, the differentially expressed genes have to be identified and their log fold-change calculated. Then one can apply the method with `spia` function on the data with EntrezGene identifiers. The human pathways from

other databases are available through the implementation from the `graphite` package (function `runSPIA`). However, a pre-processing step is required. In this step, the pathways are transformed into a SPIA-specific format and the node identifiers are converted into EntrezGene IDs. In our package we have incorporated a more general converting function and the user can also obtain a gene-level net perturbation accumulation - a measure of the importance of a gene in the topology. The entry genes are assumed to be the most important (if they are differentially expressed, the expression of the whole pathway is perturbed).

The last of the methods implemented within the R/Bioconductor framework is Clipper. It is available through two packages: `clipper` and `graphite`. The method constists of two steps: (i) first, the differential expression of a pathway is assessed, (ii) then, the pathway topology is transformed into a junction tree and the portions of the tree which are mostly associated with phenotype are identified. These two steps are implemented as two functions in `clipper` (`pathQ` and `easyClip`). However the implementation in `graphite` employs only `easyClip` function neglecting the first step. We designed a new function that performs both steps of the algorithm in a single call.

In all of the imported and adjusted implementations we preserved the possibility to modify method parameters. We also added, when appropriate, an additional parameter specifying how should be the undirected interactions oriented. The user can choose whether an edge is oriented in both directions or only in one according to the order of the nodes.

We de-novo implemented three methods: TAPPA, PWEA, PRS, for which there was no implementation available within R framework. The PRS and PWEA are implemented in MATLAB and C++ respectively and these tools are discussed in the section 3.3. Our de-novo implementations are settled for pathway topologies from `graphite` package where one node is represented by only one gene or protein. Both PWEA and PRS methods incorporate a permutation-based test in order to assess the statistical significance of the pathway score. Considering the computational complexity of this approach we parallelized the crucial step of the PWEA method (application of the differential expression analysis by moderated t-test from `limma` package). In addition, the function for obtaining the number of the differentially expressed genes in PRS algorithm was implemented in C++ via `Rcpp` package.

While several methods (TopologyGSA, DEGraph, Clipper and TAPPA) work directly with normalized gene expression values, others (SPIA, PRS and PWEA) use the result of differential gene-expression analysis with or without application of significance thresholds to obtain the list of differentially expressed genes (Figure 5). With respect to this, all the methods were adapted also for a simple use of RNA-Seq count data. First, we employed pre-processing step for RNA-Seq normalization, with a selection of two best performing methods TMM [11], DESeq [2], as compared in Dillies et al. [4] and regularized log transformation from `DESeq2` package which effectively removes the mean-variance relationship known in RNA-Seq data. Second, we added methods for RNA-Seq differential gene expression analysis (from `limma` and `DESeq2` packages).

References

[1] M. Al-Haj Ibrahim, S. Jassim, M. A. Cawthorne, and K. Langlands. A topology-based score for pathway enrichment. *J Comput Biol*, 2012.

[2] S. Anders and W. Huber. Differential expression analysis for sequence count data. *Genome Biology*, 11(10):R106, 2010.

[3] C. Bron and J. Kerbosch. Algorithm 457: Finding all cliques of an undirected graph. *Commun. ACM*, 16(9):575–577, Sept. 1973.

[4] M.-A. Dillies, A. Rau, J. Aubert, C. Hennequet-Antier, M. Jeanmougin, N. Servant, C. Keime, G. Marot, D. Castel, J. Estelle, G. Guernec, B. Jagla, L. Jouneau, D. Lalo, C. Le Gall, B. Schaffer, S. Le Crom, M. Guedj, and F. Jaffrzic. A comprehensive evaluation of normalization methods for illumina high-throughput rna sequencing data analysis. *Briefings in Bioinformatics*, 14(6):671–683, 2013.

[5] S. Gao and X. Wang. Tappa: topological analysis of pathway phenotype association. *Bioinformatics*, 23(22):3100–3102, 2007.

[6] J. J. Goeman and P. Bhlmann. Analyzing gene expression data in terms of gene sets: methodological issues. *Bioinformatics*, 23(8):980–987, 2007.

[7] J.-H. Hung, T. Whitfield, T.-H. Yang, Z. Hu, Z. Weng, and C. DeLisi. Identification of functional modules that correlate with phenotypic difference: the influence of network topology. *Genome Biology*, 11(2):R23, 2010.

[8] L. Jacob, P. Neuvial, and S. Dudoit. Gains in Power from Structured Two-Sample Tests of Means on Graphs. *ArXiv e-prints*, Sept. 2010.

[9] M. Massa, M. Chiogna, and C. Romualdi. Gene set analysis exploiting the topology of a pathway. *BMC Systems Biology*, 4(1):121, 2010.

[10] S. Niskanen and P. R. J. stergrd. Cliquer user's guide, version 1.0. Technical report, Communications Laboratory, Helsinki University of Technology, Espoo, Finland, 2003.

[11] M. Robinson and A. Oshlack. A scaling normalization method for differential expression analysis of rna-seq data. *Genome Biology*, 11(3):R25, 2010.

[12] A. L. Tarca, S. Draghici, P. Khatri, S. S. Hassan, P. Mittal, J.-s. Kim, C. J. Kim, J. P. Kusanovic, and R. Romero. A novel signaling pathway impact analysis. *Bioinformatics*, 25(1):75–82, 2009.

Figrues

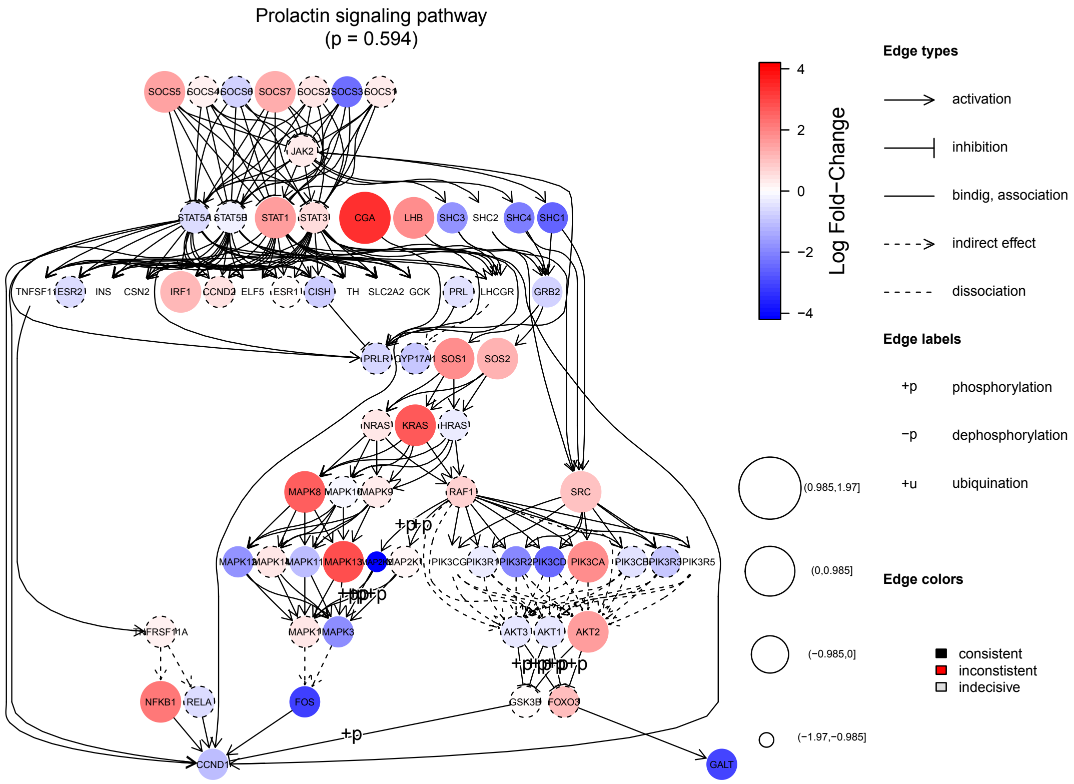

Figure 1: **Basic visualization of the results.** The filling colour of a node informs on the status of the differential expression of a gene (the user can choose between the log fold-change or the test statistic). Transparent colour is, by default, used for the nodes which were not measured in the analysis. Border of the nodes informs about statistical significance of the differential expression of the gene, where the non-significant genes are highlighted by dashed line. If a topological importance of a gene is calculated within a particular method then it is reflected in the node size. The line types and the arrowheads of the edges help to distinguish the interaction type.

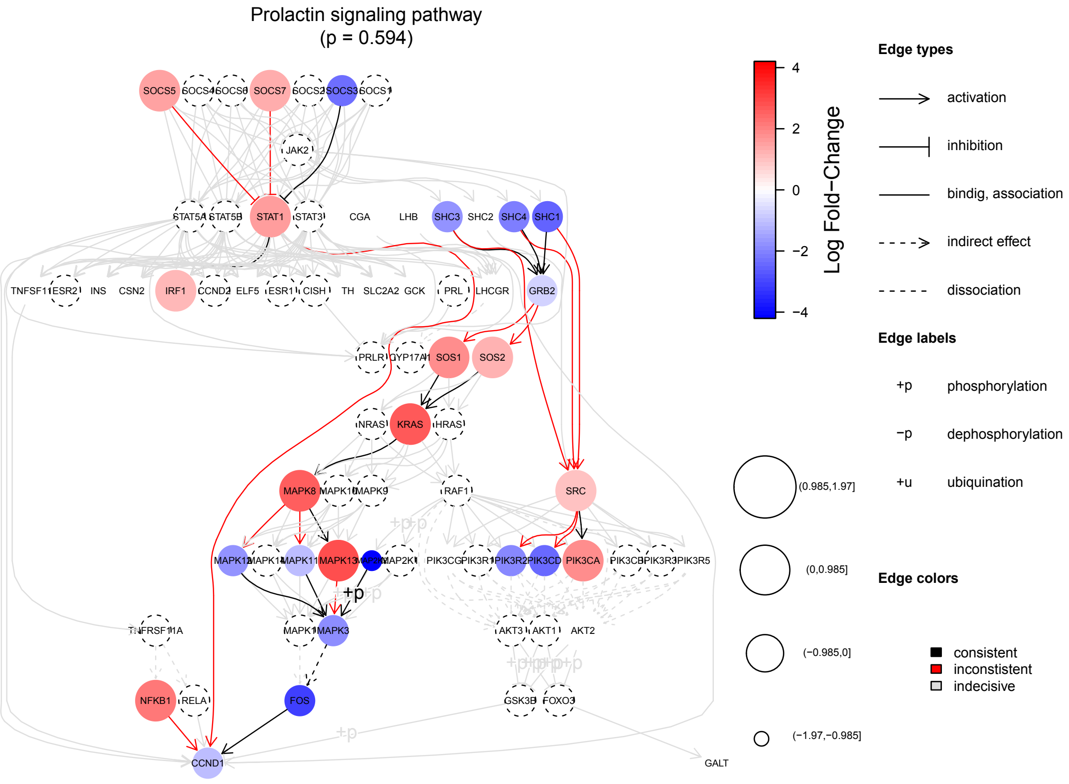

Figure 2: **Visualization of the results after the expression of interacting genes was compared with the interaction type.** Only four interaction types are relevant to the changes in the gene expression: activation, inhibition, expression, repression. Based on the differential gene expression status of the genes connected by an edge, we can divide the observed interactions into three categories: (i) expression of the nodes agrees with the interaction type (black), (ii) expression of the nodes disagrees with the interaction type (red), (iii) the interaction type is not relevant or the expression changes may be random (grey).

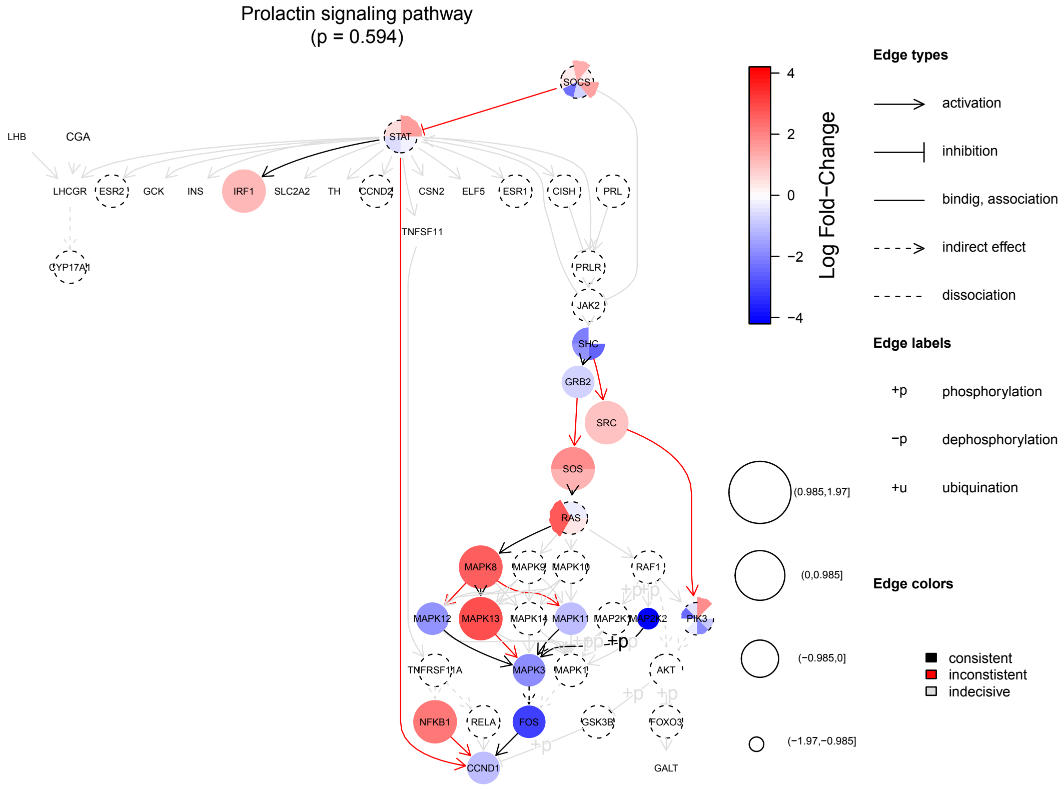

Figure 3: **Visualization of the results after merging some of the gene families into one node.** Some of the genes families present in the pathway were merged into single nodes. Those nodes are drawn as pie-chart, in which the number of slices equals to the number of gene merged. The colour, border and radius are preserved from the complete graph (Figure 2). Average log fold-change is used as representative value, when the agreement between expression and interaction type is assessed.

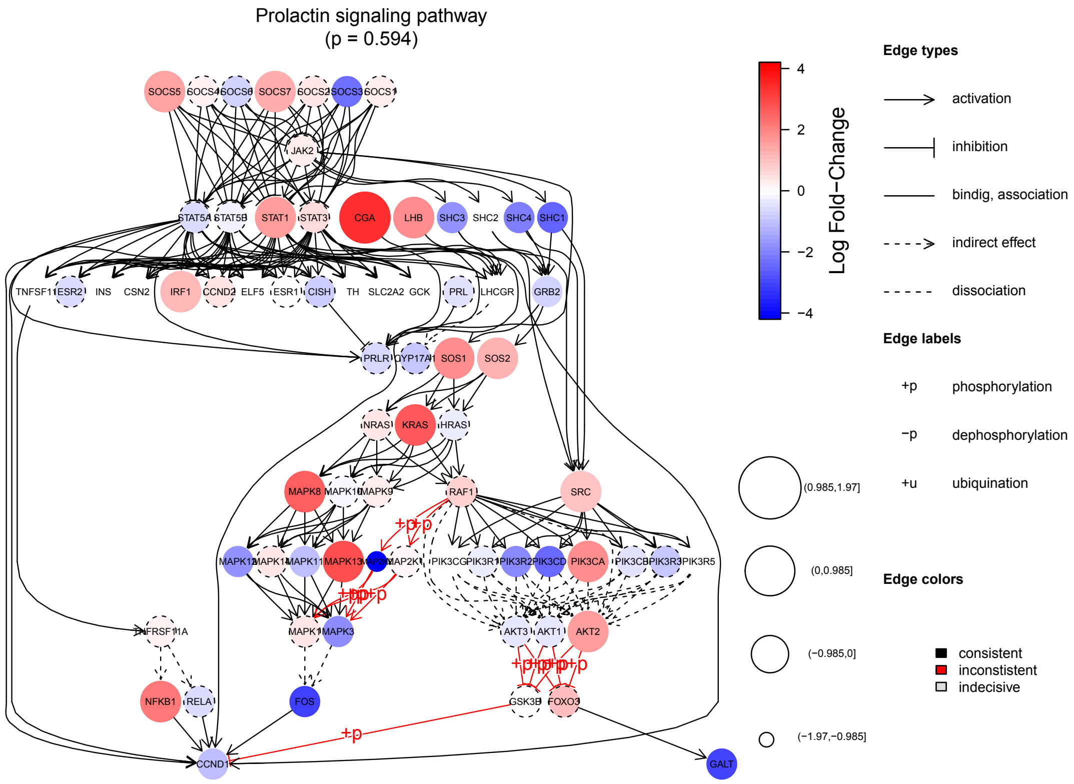

Figure 4: **Visualization of the results of the clipper method.**The filling colour of a node informs on the status of the differential expression of a gene (log fold-change). Transparent colour is, by default, used for the nodes which were not meassured in the analysis. Border of the nodes informs about statistical significance of the differential expression of nodes. The non-significant genes are highlighted by dashed line. The red lines highlight the significant cliques. Note, that the method analyses a modified topology, therefore the exact cliques can not be seen.

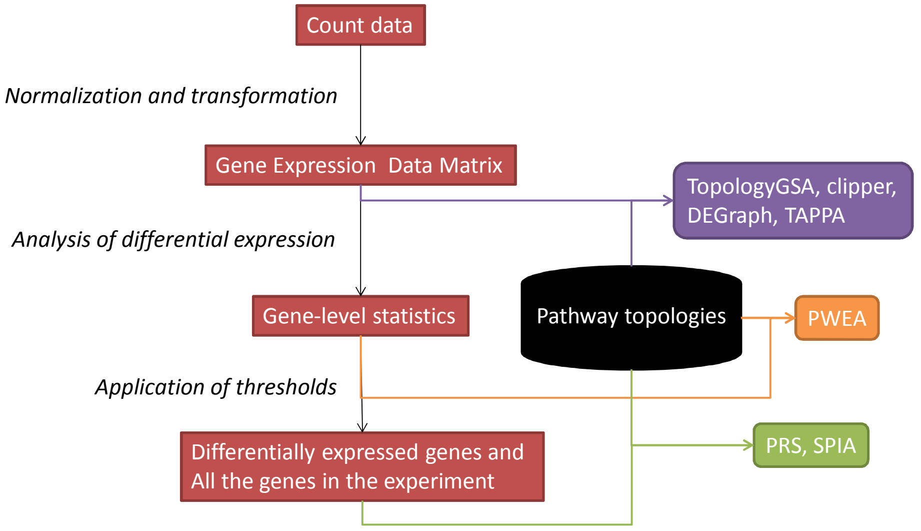

Figure 5: **Schema of a processing pipeline** The red boxes refer to the outputs from regular analysis of differentially expressed genes and possible inputs for topology-based pathway analysis. Arrows indicate the processing pipeline of each of the methods implemented in the package.
